# Adaptation of the Australian Palliative Care Phase concept to the German palliative care context: a mixed-methods approach using cognitive interviews and cross-sectional data

**DOI:** 10.1186/s12904-021-00825-z

**Published:** 2021-08-14

**Authors:** Eva Lehmann, Farina Hodiamont, Mirjam Landmesser, Carina S. Knobloch, Friedemann Nauck, Christoph Ostgathe, Bettina Grüne, Claudia Bausewein

**Affiliations:** 1 Department, of Palliative Medicine, University Hospital, LMU Munich, Marchioninistr. 15, 81377 Munich, Germany; 2 Department of Palliative Medicine, University Medical Center Goettingen, Goettingen, Germany; 3 Department of Palliative Medicine, Universitätsklinikum Erlangen, CCC Erlangen–EMN, Friedrich-Alexander-Universität Erlangen-Nürnberg (FAU), Erlangen, Germany

**Keywords:** Palliative care phases, Palliative care, Cognitive interviewing, Inter-rater reliability, Reproducibility of results, Outcome measurement, Quality of healthcare

## Abstract

**Background:**

Palliative care phases (stable, unstable, deteriorating, terminal and bereavement) are routinely used in Australia and the UK to describe the clinical situation of patients and their families and to evaluate the associated care plan. In addition, it serves as a benchmark developed by the Australian Palliative Care Outcome Collaboration (PCOC) and is used nationwide for comparisons between services. In Germany, the concept is not used consistently due to various translations. Furthermore, there is no nationwide systematic approach to routinely assess clinical outcomes in palliative care.

The study aims to develop a German version of the palliative care phase definitions by adapting them culturally, and to examine the inter-rater reliability of the adjusted definitions with healthcare professionals.

**Methods:**

Mixed-methods approach: Cognitive interview study using ‘think aloud’ and verbal probing techniques and a consecutive multi-center cross-sectional study with two clinicians independently assigning the phase definitions. Interviewees/participants were selected through convenience and purposive sampling in specialist palliative care inpatient units, advisory and community services and in three specialist palliative care units with doctors, nursing staff and allied health professionals.

**Results:**

Fifteen interviews were conducted. Identified difficulties were: Some translated terms were 1) not self-explanatory (e.g. ‘family/carer’ or ‘care plan’) and (2) too limited to the medical dimension neglecting the holistic approach of palliative care. (3) Problems of comprehension regarding the concept in general occurred, e.g. in differentiating between the ‘unstable’ and ‘deteriorating’ phase. Inter-rater reliability was moderate (kappa = 0.44; 95% CI = 0.39–0.52). The assignment of the phase ‘deteriorating’ has caused the most difficulties.

**Conclusion:**

Overall, the adapted palliative care phases are suitable to use in the German specialist palliative care setting. However, the concept of the phases is not self-explanatory. To implement it nationwide for outcome measurement/benchmarking, it requires further education, on-the-job training and experience as well as the involvement of healthcare professionals in implementation process. For the use of international concepts in different healthcare systems, a deeper discussion and cultural adaptation is necessary besides the formal translation.

**Supplementary Information:**

The online version contains supplementary material available at 10.1186/s12904-021-00825-z.

## Background

The care of seriously ill patients by specialist palliative care may become necessary if the patients’ situation proves to be complex and thus particularly resource-intensive [1]. The extent of the complexity of a care situation is determined by the intensity of individual symptom burden and/or psychosocial, spiritual or ethical problems of the patients as well as their simultaneous occurrence and interaction [2]. Studies from Australia and UK use clinical assessment tools to describe the complexity of the patient’s situation, which map the areas of severity of symptoms and problems, performance of activities of daily living and functional status, as well as the burden on relatives [2, 3]. Clinical assessment tools are standardised and validated instruments that provide the palliative care team with relevant information about the condition/situation of patients and their relatives. Either the assessment tools are completed by the patients themselves, or they are assessed by the treating palliative care team [4–6]. Therefore, the use of assessment tools influences the identification of needs, the monitoring of symptoms and the implementation of clinical interventions. Furthermore, it influences the communication between patients and doctors and leads to an improvement of patient outcomes through person-centred care [4, 7–9].

The concept ‘Palliative Care Phase’ is a clinical assessment tool which is increasingly in use since its development by the Australian Association for Hospice and Palliative Care in 1993 [10]. It was first tested and revised in 1994 [11] and for a second time in 2011 [12]. The concept includes five phases: ‘stable’, ‘unstable’, ‘deteriorating’, ‘terminal’ and ‘bereavement’. They describe the clinical situation of patients and their families as well as related care needs and the suitability of the current care plan. The concept is based on the principle that patients and their carers form the unit of care. Palliative care phase focuses on individual needs, goals and priorities rather than on the disease itself [12]. A phase changes when the existing care plan is no longer effective. Palliative care phases do not have a linear trajectory due to the unpredictable progression of incurable diseases and patients’ individual goals and needs. Therefore, phases can alternate and occur several times [12, 13]. In inpatient care in Australia, an average of two phases per patient was reported [14, 15].

Palliative care phase are significantly associated with functional status and have been proven to be an indicator of palliative needs: mean function was lowest in the terminal phase and highest in the stable phase; mean pain was highest in the unstable phase, family and carer support needs were lowest in the stable phase [13, 16]. Due to the strong association with resource use, they play a key role in the Australian casemix classification [2, 17]. Additionally, palliative care phase is embedded in nationwide routine clinical outcome measurement of specialist palliative care in Australia (since 2005) [12] and the UK (since 2013) [18]. Within these routine assessments, it has been shown that palliative care phase has benefits in everyday care. It provides a common language in the multi-professional team, facilitates care planning, serves to prioritise treatment and, thus, supports clinical decision making [16]. Palliative care phase is also used for monitoring and assurance of quality of care. The duration of the unstable phase is considered to be a quality indicator for palliative care services [12–14, 19]. Moreover, it is one of the national benchmarks developed by Palliative Care Outcome Collaboration (PCOC) since 2009 and is used for comparisons between services to develop areas for improvement and support services in improving their practice [16, 20].

For further quality improvement and to facilitate international benchmarking, a uniform approach regarding outcome measurement across palliative care services is needed. De Wolf-Linder et al. (2019) recommend using palliative care phases as the temporal indicator for these measurements in specialist palliative care. It is seen as a key element providing information on the severity and urgency of patient needs, but needs to be further improved in terms of education, context and consistency [19].

In Germany, the national guideline on palliative care for patients with incurable cancer advices that generalist or specialist palliative care has to be involved in the treatment depending on the complexity of the patient’s needs [21]. However, no evidence based system exists to differentiate the complexity of patients’ needs and care demands [22]. Moreover, there is no nationwide systematic approach to routinely assess clinical outcomes in palliative care. Additionally, there is an increasing demand for high quality care and monitoring of resource use by healthcare insurances and service providers. Palliative care phase seems to be the appropriate instrument to assess needs as well as to provide information on resource use and quality of care.

In Germany, routine outcome measurement in the clinical setting is used in some services but is not yet widely implemented. Similarly, few services use the palliative care phases both in the inpatient and home care setting. However, no data on palliative care phases or research on phases is available in Germany. Therefore, the aim of this study was to develop a culturally adapted German version of the Australian palliative care phase definitions. The objectives were i) to gain a deeper understanding of the meaning and comprehensibility of the translated phase definitions in different specialist palliative care settings in Germany and ii) to examine the inter-rater reliability of the developed instrument in German palliative care.

## Methods

### Design

Sequential mixed-methods design:

i) Cross-sectional multi-centre qualitative interview study using several rounds of cognitive interviews.
ii) Multi-centre cross-sectional study, pairs of clinicians rating patients independently applying the revised definitions of palliative care phase.

The COREQ checklist was applied to the first part of the study and the STROBE statement to the second part [23, 24] (Additional file 1).

### Cognitive interviews

#### Setting and participants

To include a variety of views and experiences, convenience and purposive sampling was based on a sampling frame (Additional file 2) including professional groups (doctors, nursing staff and allied health professionals), gender and setting [25]. Participants were recruited personally, by email or phone in all specialist palliative care settings: palliative care unit, palliative care advisory team, and specialist palliative home care.

#### Data collection and analysis

The Australian phase definitions were translated independently and compared and revised by two research associates. To evaluate how palliative care phases are understood and where difficulties may arise we used cognitive interviewing, that is usually applied to evaluate the understanding of questions in surveys [26] We developed an interview guide (Additional file 3) according to Beatty and Willis which contained instructions to think aloud technique as well as probing questions [27] The interviewees were asked to ‘think aloud’ while reading each phase definition out loud. Afterwards, they were asked what they understood and how they interpreted it [26] Specific questions were asked about the understanding of individual words/statements to generate more verbal information. Additional probing questions could be addressed, to explore verbal or non-verbal expressions such as hesitation or irritation [27].

The first two interviews were used for pilot testing the interview guide, which was approved. The following interviews were conducted iteratively in three rounds, as recommended [28]. Interviews were audio recorded. Audio records were transcribed verbatim and analysed using a systematizing qualitative analysis approach following Knafl and colleagues: They use an item-by-item analysis with a focus on how participants understood the survey questions and were able and willing to respond. Results supported decision-making for keeping, deleting or changing terms or phrases [26]. Accordingly, terms and phrases that were understandable and consistently interpreted by all participants were kept, others were modified or deleted. Data analysis was also conducted in three rounds. In order to standardise the analysis, EL and BG deductively developed a coding scheme, based on the research questions and the interview guide. The revision of the phase definitions was made (by EL, BG and CB) to be tested in the next round. The interview guide was adapted accordingly. Due to less substantive remarks and more layout and formalities in the third round, data saturation was assumed and the phase definitions were revised finally. 20% of all material was double coded. The qualitative data management software MaxQDA was used to support analysis. In total, between January and April 2020, ten interviews were conducted in a place chosen by the participants and five by phone due to the COVID-19 pandemic. In the case of interviews conducted by phone, the phase descriptions were sent to the interviewees in advance by email so that they had them at hand. The interview process had otherwise not changed.

### Cross-sectional study

The approach of the second part of the study is following the work of Masso et al. [12] in Australia.

#### Setting and participants

The study involved three different specialist palliative care units in university hospitals in Germany. None of the services routinely collected palliative care phases prior to the study. Only one service collected data on palliative care phases sporadically for over 2 years. The concept and an accompanying manual were presented to all services with video tutorials. All members of the multi-professional teams were invited to participate in the study (doctors, nurses, allied health professionals).

#### Data collection

Two participants were asked to independently assign the palliative care phase to the same patient. The bereavement phase was excluded as it is clear to assign. Assignments had been made twice a week for all inpatients with at least two days apart, e.g. on Tuesday and Friday after the ward round/regular team meeting without any prior communication on palliative care phases. The independent assignments of one patient had to be completed within two hours. Multiple assignments of one patient on separate days were possible. These were connected via a patient-ID. Furthermore, indicators of acceptability were assessed with the following additional questions (on a 5-point scale from 1–5):

1. How appropriate is the definition of the assigned phase to describe the current situation of this patient? (1 ‘not appropriate’ to 5 ‘very appropriate’)
2. How difficult was it for you today to assign a phase to this patient? (1 ‘very difficult’ to 5 ‘very easy’)
3. How familiar are you with this patient and his/her situation? (1 ‘unfamiliar’ to 5 ‘very familiar’)
4. What other information about the assignment of the palliative care phase would you like to give us?

The comments in question 4 were analysed to identify uncertainties in the assignments. To describe the patient population, the following characteristics were collected: age, sex and the diagnosis (malignant or non-malignant). Professionals were asked about sociodemographic characteristics and clinical experience: age group, profession, sex, length of clinical experience in specialist palliative care and experience in assigning palliative care phases. These information were not linked with the assignments and only used for describing the sample. All participating services designated a responsible person for data collection who ensured adherence to the study protocol. Data collection took place from June to October 2020.

#### Analysis

For inter-rater reliability Cohen's Kappa was calculated. According to Landis and Koch (1977), kappa should be 0.61–0.80 in order to assume a substantial agreement [29]. The sample size is determined based on (1) an assumed level of agreement of k = 0.67, which corresponds to the observed k in the Australian study [12], (2) the probability that professionals can observe and assess the individual phases (the prevalence of the individual phases was taken from the Australian PCOC organisation's National Report [30] published in September 2019 (stable: 25%, unstable 19%, deteriorating 35%, dying 21%) and (3) a target minimum correspondence of k = 0.61, which correlates to the lower limit of a 95% confidence interval. The sample size was calculated with the package kappaSize in R (version 4.0.2). Under the given assumptions and objectives, a sample size of n = 300 phase estimates was determined. Thus, each service should assign n = 100 palliative care phases.

Furthermore, agreement was calculated in percent and a descriptive analysis of the supplementary questions was conducted, stratified by phase, agreement/lack of agreement and institution. Comments were evaluated by content analysis. Cases with missing data in phase assignments were excluded from analysis.

## Results

### Cognitive interviews

#### Sample description

Fifteen interviews with a duration of 30 to 88 min were conducted. 15/16 persons accepted the invitation. One person did not respond. Table 1 gives the demographic details of the participants.

**Table 1:** Demographic details of the participants (*n* = 15)

		***n* = 15**
Age group (years)	25—29	3
	40—49	6
	50—59	4
	60 and older	2
Sex	Female	11
	Male	4
	Other	0
Work experience (years)	Median (range)	10 (1–20)
Care setting	Palliative care unit (1)	6
	Community palliative care team (2)	4
	Palliative care advisory team (3)	5
Profession	Doctors	8
	Nurses	4
	Allied health professionals (psychologists, therapists, social workers)	3

#### Findings from the interviews and phase definition refinement

##### Round 1

The first round was characterized by comments that were substantial to a proper understanding of the concept. Interviewees viewed the translations of the terms ‘care plan’ or’plan of care’ as well as of the term ‘emergency treatment’ as inappropriate:

*‘R: (…) By a treatment plan I would primarily understand a medical treatment plan. But probably that is not what is meant, probably the treatment plan (…) is meant in such a way that it does not only include medical procedures, medication or something, but also other supporting activities.‘ #00:04:56–6# (Eva_doctor_3: 31).*

*‘I: What do you understand by emergency treatment? #00:10:01–8#*

*R: Yes, an unplanned medical treatment. (…) But this is something that is only used in a medical context, emergency treatment, but under point three it's more like a psychosocial emergency.’ #00:10:45–4# (Eva_doctor_3: 64–65).*

Both were felt to be too limited to the medical dimension of care and to neglect the holistic approach of palliative care and its four dimensions (physical, psychological, social & spiritual). For these reasons, the German translation was adapted to terms that focus on the holistic care perspective rather than the medical treatment perspective. For instance, in the case of ´emergency treatment´ it was changed to ´emergency intervention´. The selected terms were tested in the following interview round and eventually integrated into the final version.

In addition, it became clear that the translations of some terms were not self-explanatory, for example the words ‘family/carer’ or ‘family/carers circumstances’. In this context, the participants were asked what they understand by the terms ‘family/carer’ and what kind of experience they have concerning situations of relatives that may affect the care of patients. The role of the ‘family/carer’ can be fulfilled by actual family members, but also from informal caregivers, the community/neighbourhood network or nursing homes/outpatient care services. Answers regarding the circumstances possibly affecting care were for example massive burden, alienation/unresolved conflicts in the family, relatives’ own illness/accident/death, financial difficulties and overburdening of nursing homes or outpatient care services.

Furthermore, the term ‘functional status’ was perceived as too technical and it was suggested to speak of ‘general condition’, as this is often used in communication with patients:

*‘R: Functional status, that sounds as if my car is broken or DAMAGED or/ (Laughs) (…) that is a very technical term (…) I would never use the word "functional status" when talking about a patient or a relative, because with that you reduce the person a bit. #00:15:33–5#*

*I: What term do you think of alternatively or what other term would you use then? #00:15:39–1#*

*R: Well, I would maybe say general condition, I think that's more appropriate.’ #00:16:28–2# (Günther_nurse_1: 47—50)*

Another essential comment in the first cycle of interviews was that the Australian description of the bereavement phase does not comply with the procedures in the German palliative care setting:

*‘R: Yes, that closing of the patient case in the sense of support after DEATH (…) IS correct in relation to patient support. But I don't think it should be formulated so harshly (…) signal that we nevertheless also feel responsible for accompanying relatives with difficult grief work beyond death.’ #00:29:29–4# (Claudia_doctor_3: 81).*

Therefore, the description of the phase was shortened to the sentence ‘the patient has died’ and ‘case closure’. The second round was used to collect more information on the procedures in the bereavement phase in the German specialist palliative care settings.

For reasons of clarity, the interviewees noted that they would prefer a consistent use of terms such as problem, symptom and crisis rather than using terms synonymously.

##### Round 2

Round two was used to verify the changes made after round one. All were assessed to be appropriate by those interviewed and were therefore retained. Furthermore, it was determined by collecting information about the bereavement phase which was reduced to basic information (see round 1). Participants wondered why relatives were not mentioned in this phase and stressed the importance of mentioning them due to the need of individual offers to support them in coping with grief.

*‘R: So deceased: grief counselling. The patient has passed away. And closure of the case in the care setting. (…) The relatives no longer appear now. (…) I miss them now.’ #00:11:42–6# (Inge_alliedhealth_1: 89—91)*

##### Round 3

In the last round, fewer substantive remarks were made to the definitions. Instead, it was more about layout and formalities. One interviewee recommended to change the headings ‘Start’ and ‘End’ of the phase definitions into ‘Phase’ and ‘Phase change’ as these headings correspond much better to the subsequent texts:

*‘R: Well, I would rather say, not beginning, but this is the phase, we are in the middle of it, what does phase ‘stable’ mean, what does phase ‘unstable’ mean. And then I wouldn't write end, but transition into other phases, and then describe that more concretely." #00:02:16–9# (Konrad_doctor _2: 34).*

Other layout and formal recommendations were underlining important key words to emphasize them or using arrows to highlight the need for action in relation of a phase change.

Overall, interviewees had some difficulties in the comprehension of the overall concept. This was confirmed in particular by difficulties in differentiating between the phases ‘unstable’ and ‘deteriorating’. Interviewees focused too much on patients’ status rather than on the suitability of the care plan and whether the symptom/problems had been anticipated which means phase ‘deteriorating’ or unexpected which means phase ‘unstable’.

*‘R: I find that difficult, this distinction, for me now as a conclusion, unstable, when suddenly something changes, when suddenly a change occurs and deteriorating, well, increasingly, so to speak, it goes down bit by bit.’ #00:36:08–6# (Hannah_alliedhealth _1: 171).*

These uncertainties demonstrated the relevance to emphasize the role of the care plan.

Interviewees raised the question whether changes in the circumstances of the family/carer alone are sufficient for a change of the palliative care phase. Therefore, it needs to be emphasised that according to the concept of the patient and his/her family/carer as a unit of care, palliative care phases should also be allocated corresponding to the needs of the family/carer. The only exception is the terminal phase. Even if the care plan urgently needs to be adjusted or the family/carers are increasingly burdened, the phase remains ‘terminal’.

It emerged in all three rounds that the single page of phase definitions and some terms are not self-explanatory and need to be supplemented by broader information of the concept. Due to this, a manual for implementation and application has been developed. It is based on recurring statements of the interviewees (questions raised, given definitions etc.) as well as on the literature on palliative care phases. The revised German palliative care phase definitions can be found in the Additional file 4. The developed manual can be requested from the authors.

### Cross-sectional study

#### Clinician demographics

A total of 71 clinicians participated in the study (details see Table 2). Nine participants (15.5%) had extensive work experience in palliative care of more than 15 years. More than half (50.7%) had less than five years of work experience in palliative care. Most participants were nurses (57.7%), followed by doctors (29.6%) and allied health professionals (12.7%). Overall, 37 participants (52.1%) did not have any experiences in assigning palliative care phases.

**Table 2:** Demographic details of the clinician participants (*n* = 71)

		***n* = 71**	**%**
Age group (years)	18—24	2	2.8
	25—29	5	7.0
	30—39	20	28.2
	40—49	18	25.4
	50—59	18	25.4
	60 and older	8	11.3
Profession	Doctors	21	29.6
	Nurses	41	57.7
	Allied health professionals	9	12.7
Sex	Female	51	71.8
	Male	20	28.2
	Other	0	0
Work experience in palliative care (years)	less than 5	36	50.7
	5—9	11	15.5
	10—14	15	21.1
	15 years or more	9	15.5
Experience with phase assignment	Yes	34	47.9
	No	37	52.1

#### Patient characteristics

On average, patients were 69.9 years old and the majority (77.2%) had a malignant disease. Gender distribution was almost balanced (Table 3).

**Table 3:** Patient characteristics across all in-patient palliative care units

		***n* = 373**	**%**
Age group (years)	25–54	39	10.5
	55–64	85	22.8
	65–74	106	28.4
	75–84	96	25.7
	85 +	47	12.6
Sex	Female	187	50.1
	Male	186	49.9
	Other	0	0
Diagnosis	Malignant	288	77.2
	Non-Malignant	85	22.8

#### Inter-rater reliability

Of all double assignments of palliative care phases, 237 (63.5%) matched and 136 (36.5%) did not match. Most disagreements (78.7%) were referred to the combinations stable/deteriorating, stable/unstable and unstable/deteriorating (Table 4). Seventy five point one percent of all assignments were completed within a time interval of 2 h.

**Table 4:** Characteristics of ratings by two clinicians (*n* = 373)

Rating	Phase	n	%
Agreed assignments	stable	142	37.9
	unstable	10	2.7
	deteriorating	45	12.0
	terminal	40	10.7
Disagreed assignments	stable—deteriorating	49	13.1
	stable—unstable	35	9.3
	stable—terminal	9	2.4
	unstable—deteriorating	23	6.1
	unstable—terminal	5	1.3
	deteriorating—terminal	15	4.0

In total, the kappa value was 0.44 (95% CI: 0.35–0.62) representing a moderate level of agreement (Table 5). Palliative care in-patient units differed in their level of agreement: unit 1 and unit 3 achieved a moderate agreement, the agreement in unit 2 was fair.

**Table 5:** Rater agreement by in-patient palliative care unit

Unit	Number of assignments	Percentage of actual agreement	Kappa	95% confidence interval	Strength of agreement
1	185	67.0	0.48	0.37—0.59	Moderate
2	80	51.2	0.29	0.13—0.45	Fair
3	108	66.7	0.49	0.39—0.52	Moderate
Overall	373	63.5	0.44	0.35—0.62	Moderate

#### Acceptability

In Table 6, the indicators of acceptability are listed. Higher scores for ‘degree of fit’ and ‘ease of assignment’ were found for the phases ‘stable’ and ‘terminal’ than for the phases ‘unstable’ and ‘deteriorating’. In addition, the scores for ‘degree of fit’ and ‘ease of assignment’ were higher for agreed ratings than for non-matched ratings. Familiarity with patient’s situation was low in general, but also higher for the phases ‘stable’ and ‘terminal’.

**Table 6:** Degree of fit, ease of assignment and familiarity with patient’s situation by palliative care phase and match/mismatch

Palliative care phase	Degree of fit		Ease of assignment		Familiarity with patient’s situation	
	Mean	SD	Mean	SD	Mean	SD
Stable	3.24	0.84	4.22	0.91	3.00	1.03
Unstable	2.47	1.23	3.37	1.06	2.53	1.06
Deteriorating	2.86	0.90	3.70	0.97	2.69	1.12
Terminal	3.61	0.77	4.46	0.82	2.92	1.09
Match	3.29	0.86	4.23	0.89	2.94	1.05
Mismatch	2.83	10.4	3.70	1.06	2.72	1.11

#### Comments by raters

The analysis of the 42 comments given, indicated some challenges in assigning palliative care phases. However, few comments were notable (Table 7). Many comments provided by participants indicated that the concept was understood by the participants.

**Table 7:** Comments by raters

Comment	Phase	Comments of the clinicians
1	Stable	Support during the dying process, nevertheless stable phase
2	Unstable	Expected, but only potential bleeding complication, therefore unstable
3	Unstable	Fluctuating course, but problems were anticipated. Therefore, not unexpected, but still unstable
4	Deteriorating	Caring relatives are burdened applies but does not apply to patient care
5	Deteriorating	Line between deteriorating and dying unclear. Here, for example, the patient’s general condition has steadily deteriorated and death is likely within days

## Discussion

In this study, we have developed a culturally adapted version of the Australian concept of palliative care phase and determined the inter-rater reliability of the revised German definitions. In Australia, the palliative care phase concept is very well established in specialist palliative care and is used nationwide [12]. However, in order for it to be used in other countries and their healthcare systems, it needs to be adapted to the prevailing conditions.

The results of cognitive interviewing showed that the formal translation of the phases was not fitting adequately to wording and procedures in the German specialist palliative care setting, e.g. the description of the bereavement phase or the need to use terms consistently. Moreover, some terms were not self-explanatory for the professionals and the single page of phase definitions was insufficient to convey a comprehensive understanding of the concept. These problems in understanding the concept became particularly apparent in respect of the phases ‘deteriorating’ and ‘unstable’. Interviewees focused solely on patients’ condition rather than on the suitability of the care plan and whether the symptom/problems had been anticipated or not. The observed uncertainties with single terms as well as with the overall concept emphasized the need to complement the phase definition with further information in an additional manual. Overall, the results confirmed the need to adapt the formal translation of palliative care phases to the cultural context of the German specialist palliative care setting and consider setting specific wording/language as well as educational needs of professionals. The necessity of cultural adaptations was also reported by other studies that translated patient reported outcomes [28, 31–34].

The inter-rater reliability showed a moderate degree of agreement (Kappa = 0.48) within participating centres. Compared to the Australian study [12] (kappa = 0.67) the agreement was lower. Most mismatches were, as in Australia, stable/deteriorating, stable/unstable and unstable/deteriorating combinations. The overall mean ‘degree of fit’ of 3.12 was comparable to the Australian data (mean ‘degree of fit’ of 3.28) and indicated that the developed phase definitions are appropriate to apply in Germany. In addition, all scores for ‘degree of fit’ and ‘ease of assignment’ were higher for matched ratings than for non-matched ratings. Furthermore, the phases ‘unstable’ and ‘deteriorating’ had lower values (2.47 and 2.86) and were therefore more difficult to assign than the phases ‘stable’ and ‘terminal’ (3.37 and 3.7). The higher number of mismatches, when these phases were included, confirms this. It moreover indicates that especially ‘terminal’ and ‘stable’ phases were easier to assign and the phase description fitted better with the patient’s situation. This is not surprising, as these phases are more obvious and thus easier to assign. Looking at the comments given after the assignment, the first and fifth indicate that these participants had difficulties in understanding the phase ‘terminal’ as they disregarded that the terminal phase overrules the other phases. Both, the second and third comment indicate that the participants have understood the concept. Nevertheless, they assigned the phase ‘unstable’, although they wrote that problems had been anticipated. These are aspects that must be taken into account for the education and implementation process.

When comparing our data to the Australian data, it must be taken into account that the preconditions in both studies were different. In Australia, the palliative care phases had been introduced in 1993 and since PCOC was founded in 2005, they are firmly established and accepted across the country [10, 11]. Furthermore, 55% of the participants in Australia had more than 15 years of work experience in palliative care. The same percentage also participated in a PCOC education workshop and on the job training [12] In our study, only 12.7% of participants had more than 15 years of work experience and about half (50.7%) less than five years. In addition, more than half of the participants (52.1%) had no experience in assigning palliative care phases. This emphasizes that training, practice and experience is essential to apply the concept correctly and adequately. Australian data confirms this: in the first study on inter-rater reliability in 1996, the level of agreement was 0.736 and the associated kappa statistic was 0.52, which was lower than in the study conducted in 2015 [11].

Our data also give some insights that factors beyond experience might be associated with the assignment of palliative care phases and, thus, the level of agreement. Unit 2 had greater disagreements in allocating the phases than the other two units although the characteristics of the participants including the experience with palliative care phase and patient population did not differ significantly. One reason for this could have been the lower number of cases. The analysis of the comments also indicated that some participants potentially might have understood the concept but did not follow it. This could be referred to non-acceptance of the concept and its purpose and benefits. To increase the acceptance of outcome measures, Antunes and colleagues recommend explaining the benefits in more detail, giving insight on how the instrument is constructed and discussing how the application can be handled in everyday work [35]. In this case it might be helpful to provide practice sessions and feedback, especially for the unstable and deteriorating phase. For a successful long-term implementation, these factors must be taken into account and appropriate teaching materials must be developed and included.

### Strengths and limitations of the study

The strength of this study was the inclusion of multiple professions in the cognitive interviews and in the determination of the inter-rater reliability, which allowed us to gain comprehensive insights. Moreover, the interviews were conducted in several specialist palliative care settings, i.e. inpatient units, consultative and community services. Due to the integration of three settings, an instrument was developed that applies to all specialist palliative care settings in Germany. The method of cognitive interviewing has proven to be very suitable, as it reflected the views and experiences of the clinical experts and, in addition, revealed open questions concerning the concept. There are also some limitations in this study. Due to limited resources, there was no backward translation and no wider psychometric testing like validity and test–retest reliability. However, the results demonstrate that cognitive interviewing was the more important step as cultural adaptations were proven to be essential. Although the setting of Part ii) was limited to inpatient care, three units were recruited to participate, yet we cannot provide information on how it is in other settings. Unfortunately, we do not know whether the participants used the manual provided for the second part of the study and whether it was useful for them. Nor could we ascertain how the concept was appreciated, accepted, and internalised by the participants in the units. This may have varied from one unit to another.

## Conclusion

Overall, the palliative care phases are suitable to use in the German specialist palliative care setting. The phases of the patients’ conditions can also be identified, are applicable and a moderate inter-rater agreement has been reached. In view that outcome measures/benchmarking will play a bigger role in the future, the concept is a useful tool for this purpose. It thus contributes to further development and improvement of palliative care in Germany. However, it became clear that the concept is not easy to understand. It requires education, on-the-job training, and experience as well as the involvement of healthcare professionals to reduce resistance in order to implement it nationwide. As the implementation of new outcome measurement tools can encounter resistance in practice [36], it would be important for future research to also evaluate the process of implementation in Germany and internationally and how, for example provided teaching materials are used.

## Supplementary Information

**Additional file 1.** COREQ Reporting Checklist

**Additional file 2.** Sampling frame

**Additional file 3.** Interview guides

**Additional file 4.** German version of palliative care phase definitions

**Additional file 5.** Australian Palliative Care Phase definitions.

## Data Availability

The datasets generated and/or analysed during the current study are not publicly available due [data agreement reasons: interview manuscripts cannot be anonymised entirely, the interviewees’ anonymity would not be guaranteed] but are available from the corresponding author on reasonable request.
