# Supplementary material for: Adaptation of the Australian Palliative Care Phase concept to the German palliative care context: a mixed-methods approach using cognitive interviews and cross-sectional data
Source: BMC Palliat Care. 2021 Aug 14;20:128. doi: 10.1186/s12904-021-00825-z (PMC8364299; doi:10.1186/s12904-021-00825-z)
Supplement: Supplementary file 1 — Additional file 1. COREQ Reporting Checklist [file 12904_2021_825_MOESM1_ESM.docx]

**COREQ Reporting Checklist**

| Domain 1: Research team and reflexivity | |
| --- | --- |
| Personal Characteristics | |
| 1. Interviewer/facilitator | Eva Lehmann (EL) and Bettina Grüne (BG) conducted the interviews. |
| 1. Credentials | EL - Master of Science, BG - Dr. rer. biol. hum. |
| 1. Occupation | Research Associate |
| 1. Gender | Female |
| 1. Experience and training | Not reported due to space limitations: EL - Master Degree in Health Service Research, research experience in the context of student projects, BG - Master Degree in Public health, conception and realization of one qualitative interview study and several quantitative studies. |
| Relationship with participants | |
| 1. Relationship established | Not reported due to space limitations: The research team and the interviewees did not know each other, there was no established relationship. |
| 1. Participant knowledge of the interviewer | Not reported due to space limitations: The interviewees did only know that the research team is conducting research on the topic. |
| 1. Interviewer Characteristics | Not reported due to space limitations: The interviewers have a research interest in outcome measurement in palliative care and therefore, the adaptation of international concepts to other healthcare systems and to Germany is relevant. |
| Domain 2: Study design | |
| Theoretical Framework | |
| 1. Methodological orientation and theory | Stated in the methods sections “design” and “data collection and analysis”. Data were analysed using a systematizing qualitative analysis approach. |
| Participant selection | |
| 1. Sampling | Stated in the methods section, “setting and participants”, purposive and by suggestions of the project lead. |
| 1. Method of approach | Not reported due to space limitations: An invitation letter to take part in the study was sent by email by the project lead (Prof. Bausewein). In case of acceptance, a date for a face-to-face/phone interview was arranged by either email or telephone. |
| 1. Sample size | Reported in the first paragraph of the results section and table 1. |
| 1. Non-participation | Reported in the first paragraph of the results section. |
| Setting | |
| 1. Setting of data collection | Reported in the methods section “setting and participants”. |
| 1. Presence of non-participants | Not reported due to space limitations: No one else was present besides the participants and the interviewer. |
| 1. Description of Sample | Reported in the first paragraph of the results section and table 1. |
| Data Collection | |
| 1. Interview Guideline | The development of the interview guide is described in the methods section, “Data collection and analysis”. The translated interview guide was added as an additional file. The original interview guide (in German language) is available from the authors on request. |
| 1. Repeat interviews | Not reported due to space limitations: No repeat interview was necessary. Interviewees were asked to be contacted again in case of any uncertainties or lack of information becoming evident subsequent to the interview. The project team did not have to make use of this possibility. No technical problems occurred which could have prompted the necessity of a repeat interview. |
| 1. Audio/Visual recording | Reported in the methods section, “data collection and analysis”. |
| 1. Field notes | Not reported due to space limitations: A field-note form was filled in by the researchers after each interview, covering the following topics: **Welcome and briefing** (🡪voluntariness, openness, unexpectedness), **Interview process** (🡪openness, unexpectedness, Was anything particularly important/unpleasant/... for the interviewee? If breaks were taken, why and what happened during the breaks?), **Interview setting** (🡪What kind of room? Were there any disturbing factors (interruptions, noise, light, temperature)?), **Interviewer** (🡪What else happened after the tape was switched off?), **Interpersonal** (🡪First impression, did they already know each other, etc.?), **“Feedback on the interview**”, **During the pilot interviews/first interviews** (🡪Which questions worked well? Was something not intuitive?) |
| 1. Duration | Reported in the first paragraph of the results section “sample description”. |
| 1. Data saturation | Reported in the method section “data collection and analysis”. |
| 1. Transcripts returned | Not reported due to space limitations: Transcripts were not returned to participants. Statements and descriptions were, however, continuously confirmed by the interviewing researcher during the interview in order to guarantee the correct understanding. |
| Domain 3: Analysis and findings | |
| Data analysis | |
| 1. Number of data coders | Reported in the methods section “data collection and analysis”. |
| 1. Description of the coding tree | Only by representing the findings, due to space limitations. Available from the authors on request. |
| 1. Derivation of themes | Reported in the methods section “data collection and analysis”. |
| 1. Software | Reported in the methods section “data collection and analysis”: MaxQDA. |
| 1. Participant checking | Not reported due to space limitations: Participants were not asked to provide feedback on the findings. |
| Reporting | |
| 1. Quotations presented | Quotations were presented and identified, reported in results section |
| 1. Data and findings consistent | Yes |
| 1. Clarity of major themes | Yes, we presented the major themes. |
| 1. Clarity of minor themes | Yes, as far as space limitations permitted, we discussed minor themes, too. |

**STROBE Statement—Checklist of items that should be included in reports of *cross-sectional studies***

|  | Item No | Recommendation | Page No |
| --- | --- | --- | --- |
| **Title and abstract** | 1 | (*a*) Indicate the study’s design with a commonly used term in the title or the abstract | 1 |
|  |  | (*b*) Provide in the abstract an informative and balanced summary of what was done and what was found | 1-2 |
| Introduction | | | |
| Background/rationale | 2 | Explain the scientific background and rationale for the investigation being reported | 3-4 |
| Objectives | 3 | State specific objectives, including any prespecified hypotheses | 4 |
| Methods | | | |
| Study design | 4 | Present key elements of study design early in the paper | 4-5 |
| Setting | 5 | Describe the setting, locations, and relevant dates, including periods of recruitment, exposure, follow-up, and data collection | 6 |
| Participants | 6 | (*a*) Give the eligibility criteria, and the sources and methods of selection of participants | 6 |
| Variables | 7 | Clearly define all outcomes, exposures, predictors, potential confounders, and effect modifiers. Give diagnostic criteria, if applicable | 6-7 |
| Data sources/ measurement | 8* | For each variable of interest, give sources of data and details of methods of assessment (measurement). Describe comparability of assessment methods if there is more than one group | 6-7  as far as applicable |
| Bias | 9 | Describe any efforts to address potential sources of bias | 6 |
| Study size | 10 | Explain how the study size was arrived at | 7-8 |
| Quantitative variables | 11 | Explain how quantitative variables were handled in the analyses. If applicable, describe which groupings were chosen and why | 7 |
| Statistical methods | 12 | (*a*) Describe all statistical methods, including those used to control for confounding | 7-8 |
|  |  | (*b*) Describe any methods used to examine subgroups and interactions | Not applicable |
|  |  | (*c*) Explain how missing data were addressed | 8 |
|  |  | (*d*) If applicable, describe analytical methods taking account of sampling strategy | Not applicable |
|  |  | (*e*) Describe any sensitivity analyses | Not applicable |
| Results | | | |
| Participants | 13* | (a) Report numbers of individuals at each stage of study—eg numbers potentially eligible, examined for eligibility, confirmed eligible, included in the study, completing follow-up, and analysed | 12-13 |
|  |  | (b) Give reasons for non-participation at each stage | Not applicable |
|  |  | (c) Consider use of a flow diagram | Not applicable |
| Descriptive data | 14* | (a) Give characteristics of study participants (eg demographic, clinical, social) and information on exposures and potential confounders | 12-13 |
|  |  | (b) Indicate number of participants with missing data for each variable of interest | Not applicable |
| Outcome data | 15* | Report numbers of outcome events or summary measures | 13-15 |
| Main results | 16 | (*a*) Give unadjusted estimates and, if applicable, confounder-adjusted estimates and their precision (eg, 95% confidence interval). Make clear which confounders were adjusted for and why they were included | 13-14 |
|  |  | (*b*) Report category boundaries when continuous variables were categorized | Not applicable |
|  |  | (*c*) If relevant, consider translating estimates of relative risk into absolute risk for a meaningful time period | Not applicable |
| Other analyses | 17 | Report other analyses done—eg analyses of subgroups and interactions, and sensitivity analyses | 14-15 |
| Discussion | | | |
| Key results | 18 | Summarise key results with reference to study objectives | 15-17 |
| Limitations | 19 | Discuss limitations of the study, taking into account sources of potential bias or imprecision. Discuss both direction and magnitude of any potential bias | 17-18 |
| Interpretation | 20 | Give a cautious overall interpretation of results considering objectives, limitations, multiplicity of analyses, results from similar studies, and other relevant evidence | 18 |
| Generalisability | 21 | Discuss the generalisability (external validity) of the study results | 16-17 |
| Other information | | | |
| Funding | 22 | Give the source of funding and the role of the funders for the present study and, if applicable, for the original study on which the present article is based | 19 |

*Give information separately for exposed and unexposed groups.

**Note:** An Explanation and Elaboration article discusses each checklist item and gives methodological background and published examples of transparent reporting. The STROBE checklist is best used in conjunction with this article (freely available on the Web sites of PLoS Medicine at http://www.plosmedicine.org/, Annals of Internal Medicine at http://www.annals.org/, and Epidemiology at http://www.epidem.com/). Information on the STROBE Initiative is available at www.strobe-statement.org.
