# Supplementary material for: Adaptation of the Australian Palliative Care Phase concept to the German palliative care context: a mixed-methods approach using cognitive interviews and cross-sectional data
Source: BMC Palliat Care. 2021 Aug 14;20:128. doi: 10.1186/s12904-021-00825-z (PMC8364299; doi:10.1186/s12904-021-00825-z)
Supplement: Supplementary file 2 — Additional file 2. Sampling frame [file 12904_2021_825_MOESM2_ESM.docx]

**Additional file 2.** Sampling frame

| **Characteristics** | | **n** |
| --- | --- | --- |
| Care setting | Palliative care unit | 3-6 |
|  | Community palliative care team | 3-6 |
|  | Palliative care advisory team | 3-6 |
| Profession | Doctors | 4-6 |
|  | Nurses | 4-6 |
|  | Allied health professionals (psychologists, therapists,  social workers) | 1-3 |
| Sex | Male | Minimum 3 |
