# Supplementary material for: Adaptation of the Australian Palliative Care Phase concept to the German palliative care context: a mixed-methods approach using cognitive interviews and cross-sectional data
Source: BMC Palliat Care. 2021 Aug 14;20:128. doi: 10.1186/s12904-021-00825-z (PMC8364299; doi:10.1186/s12904-021-00825-z)
Supplement: Supplementary file 3 — Additional file 3. Interview guides [file 12904_2021_825_MOESM3_ESM.docx]

**Additional file 3.** Interview guides

**Introduction**

*The respondent is given the palliative care phase descriptions.*

- Please read the phase description aloud.
- Please say aloud any thoughts that come to your mind about each section; even the things that may seem unimportant to you.
- Please let me know if anything is unclear to you or you do not understand a section.

**General questions for understanding (can be used for all phases)**

| **Questions for understanding** |
| --- |
| - What do you understand by the section you have read? - Is there anything you did not understand about the phase description "xx"? - Are there any words that are misleading? - Is there anything that is unclear? - Did any individual words or phrases particularly catch your eye? Why? - What would you write/phrase differently? |
| **Concluding questions** |
| - Are there still aspects that we have not considered in the phase description? - Are there still aspects that you are missing in the phase description? |

**Question about the whole phase description:**

- Is there anything you would like to say about this?

**Specific queries (meaning of terms/alternative terms)**

| **Round 1** | **Round 2** | **Round 3** |
| --- | --- | --- |
| **Introduction** | **Introduction** | **Introduction** |
| - What do you understand by "holistic clinical assessment"? - Which term would fit better for you? Identifies or describes? |  |  |
| **Stable** | **Stable** | **Stable** |
| - What do you understand by treatment plan? - What should the treatment plan include?   - What do you understand by care plan?   🡪 What is the difference? Which term do you find more appropriate?   - What do you understand by the term ‘relatively stable’? - What do you think about the term needs? Is this the appropriate term?   - Would requirements be better?   - Or even needs and requirements?   🡪 Can you explain your preferences? | - What do you understand by treatment plan?   - What should the treatment plan include?   🡪 Do you think another term is more appropriate?   - What do you think about care plan? |  |
| **Unstable** | **Unstable** | **Unstable** |
| - What do you understand by emergency treatment? - Which term would fit better for you?   - quickly/rapid/fast (or a completely different one?) - What circumstances of family/carers can affect patient care? - What does ‘within days’ mean to you? | - What do you understand by emergency intervention?   🡪 Do you think another term is more appropriate?   - What circumstances of family/carers can affect patient care? |  |
| **Deteriorating** | **Deteriorating** | **Deteriorating** |
| - What do you understand by functional status?   - Is there another term that is more suitable for you? (General condition?) - What do you think about the title of this phase ‘deteriorating’?   - What title would you give to this phase? - What do you think about the title ‘expected to deteriorate’? | - What do you understand by functional status?   - Is there another term that is more suitable for you? (General condition?) - What do you think about the title of this phase ‘deteriorating’?   - What title would you give to this phase? - What do you think about the title ‘expected to deteriorate’? |  |
| **Bereavement - post death support** | **Bereavement - post death support** | **Bereavement - post death support** |
|  | - The interviews so far have shown that this is also an important phase, but one about which we know little.   - What happens after the death of a patient?   - What is the process? - What do you have to think about after the death of a patient? | - The interviews so far have shown that this is also an important phase, but one about which we know little.   - What happens after the death of a patient?   - What is the process? - What do you have to think about after the death of a patient? |
