# Supplementary material for: Adaptation of the Australian Palliative Care Phase concept to the German palliative care context: a mixed-methods approach using cognitive interviews and cross-sectional data
Source: BMC Palliat Care. 2021 Aug 14;20:128. doi: 10.1186/s12904-021-00825-z (PMC8364299; doi:10.1186/s12904-021-00825-z)
Supplement: Supplementary file 4 — Additional file 4. German version of palliative care phase definitions [file 12904_2021_825_MOESM4_ESM.docx]

**
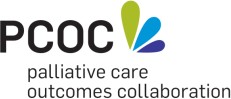
Additional file 4.** German version of palliative care phase definitions.

**Beschreibung der Palliativphasen**

| Die Palliativphase beschreibt einen klinisch bedeutsamen Zeitraum im Krankheitsverlauf von Patienten^^[[1]](#footnote-1)^^ und wird durch eine ganzheitliche Einschätzung bestimmt, die die Symptome und Probleme sowie die Bedürfnisse der Patienten und ihrer betreuenden Angehörigen berücksichtigt. Die Phasen sollen helfen, die Versorgung durch das Palliativteam zu koordinieren. | |
| --- | --- |
| **PHASE** | **PHASENWECHSEL** |
| **Stabil** | |
| - Symptome und Probleme (psychosozial und spirituell) des Patienten werden durch den Versorgungsplan angemessen kontrolliert **und** - weitere Maßnahmen zur Aufrechterhaltung der Symptomkontrolle und der Lebensqualität sind geplant   **und**   - die Situation der betreuenden Angehörigen ist relativ stabil und es sind keine neuen Probleme erkennbar. | Änderungen des Versorgungsplans sind erforderlich, da die Symptome/Probleme des Patienten zunehmen **und/oder** die Umstände/Bedürfnisse der betreuenden Angehörigen sich verändern und sich auf die Patientenversorgung auswirken.   - Dringende Änderungen/Interventionen aufgrund unerwarteter Veränderungen (🡪Wechsel in die Phase „instabil“) - Optimierungen aufgrund erwarteter Veränderungen (🡪Wechsel in die Phase „sich verschlechternd“) - Der Tod ist innerhalb von Tagen wahrscheinlich (🡪Wechsel in die Phase „sterbend“). |
| **Instabil** | |
| Eine dringende Änderung des Versorgungsplans oder eine notfallmäßige Intervention ist erforderlich, **weil**   - bei dem Patienten ein neues, unerwartetes Symptom/Problem auftritt **und/oder** - eine unerwartete, rasche Verschlechterung eines bestehenden Symptoms/Problems auftritt   **und/oder**   - die Umstände/Bedürfnisse der betreuenden Angehörigen sich unerwartet verändern und sich auf die Patientenversorgung auswirken. | Der neue Versorgungsplan und entsprechende Maßnahmen wurden erarbeitet und überprüft. Dies bedeutet nicht unbedingt, dass das Symptom/Problem vollständig abgeklungen ist,   - das Problem ist jedoch erkannt, der Versorgungsplan angepasst und die Maßnahmen beginnen Wirkung zu zeigen (🡪Wechsel in die Phase „stabil“ oder „sich verschlechternd“) **oder** - der Tod ist innerhalb von Tagen wahrscheinlich (🡪Wechsel in die Phase „sterbend“). |
| **Sich verschlechternd** | |
| Der Versorgungsplan berücksichtigt zu erwartende Symptome/Probleme des Patienten, erfordert jedoch eine regelmäßige Überprüfung und fortlaufende Optimierung, da   - sich der Allgemeinzustand des Patienten insgesamt stetig verschlechtert **und/oder** - es eine allmähliche Verschlechterung eines bestehenden Symptoms/Problems gibt **und/oder** - ein neues, aber zu erwartendes Symptom/Problem auftritt   **und/oder**   - betreuende Angehörige zunehmend belastet sind, was sich auf die Patientenversorgung auswirkt. | - Der Versorgungsplan ist geändert und der Zustand des Patienten stabilisiert sich (🡪Wechsel in die Phase „stabil“) **oder** - eine dringende Änderung des Versorgungsplans oder eine notfallmäßige Intervention ist erforderlich   **und/oder**  die Umstände/Bedürfnisse der betreuende Angehörigen verändern sich unerwartet, wirken sich auf die Patientenversorgung aus und erfordern eine rasche Intervention (🡪Wechsel in die Phase „instabil“) **oder**   - der Tod ist innerhalb von Tagen wahrscheinlich (🡪Wechsel in die Phase „sterbend“). |
| **Sterbend** | |
| - Der Tod ist innerhalb von Tagen wahrscheinlich.   **CAVE:** Auch wenn die betreuenden Angehörigen durch die Situation belastet sind und es sich auf die Patientenversorgung auswirkt, bleibt die Phase „sterbend“. | - Der Patient verstirbt (🡪Wechsel in die Phase „verstorben“) **oder** - der Zustand des Patienten ändert sich und der Tod innerhalb von Tagen ist nicht länger wahrscheinlich (🡪Wechsel in eine andere Phase). |
| **Verstorben - Trauer** | |
| - Der Patient ist verstorben.   Betreuende Angehörige werden begleitet und über Unterstützungsangebote informiert. | - Abschluss des Falls im Betreuungssetting. |

Angepasst mit freundlicher Genehmigung von: © PCOC 2014. This work is copyright. Apart from any use as permitted under the Copyright Act 1968, no part may be reproduced by any process without permission from the Palliative Care Outcomes Collaboration (PCOC)

1. Mit dem Begriff Patient sind ausdrücklich das weibliche und andere Geschlechter miteingeschlossen. [↑](#footnote-ref-1)
