# Supplementary figures and images for: Adaptation of the Australian Palliative Care Phase concept to the German palliative care context: a mixed-methods approach using cognitive interviews and cross-sectional data
Source: BMC Palliat Care. 2021 Aug 14;20:128. doi: 10.1186/s12904-021-00825-z (PMC8364299; doi:10.1186/s12904-021-00825-z)

**Additional file 5.** Australian Palliative Care Phase definitions.


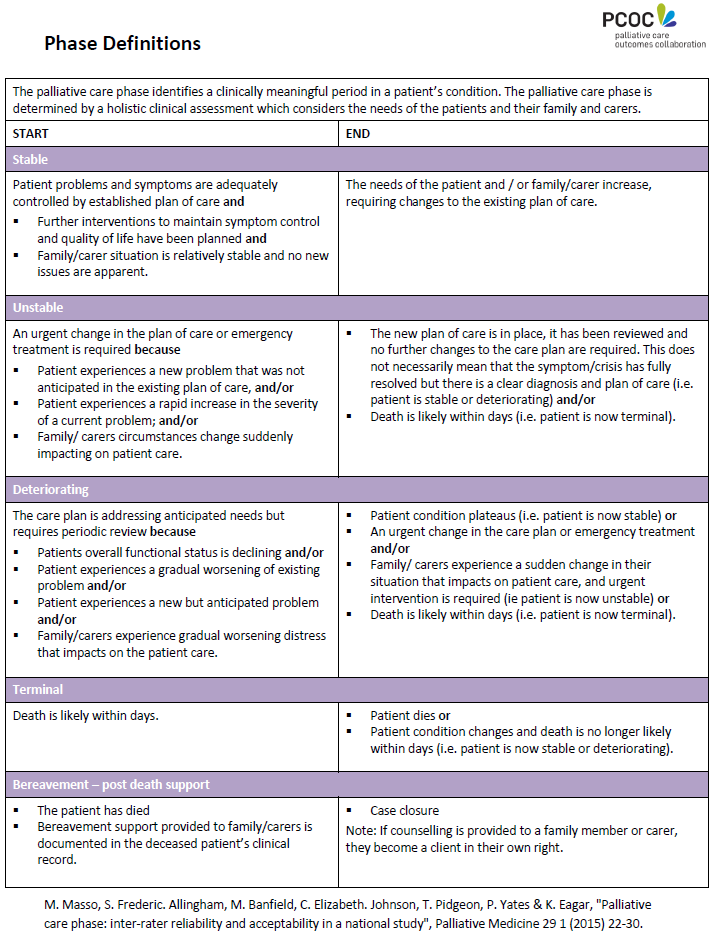

Supplement: Supplementary file 5 — Additional file 5. Australian Palliative Care Phase definitions. [file 12904_2021_825_MOESM5_ESM.docx]
